# A Hitchhiker’s Guide to Problem Selection in Carbohydrate Synthesis

**DOI:** 10.1021/acscentsci.3c00507

**Published:** 2023-07-12

**Authors:** Xavier S. Streety, Jennifer C. Obike, Steven D. Townsend

**Affiliations:** Department of Chemistry, Vanderbilt University, Nashville, Tennessee 37235, United States

## Abstract

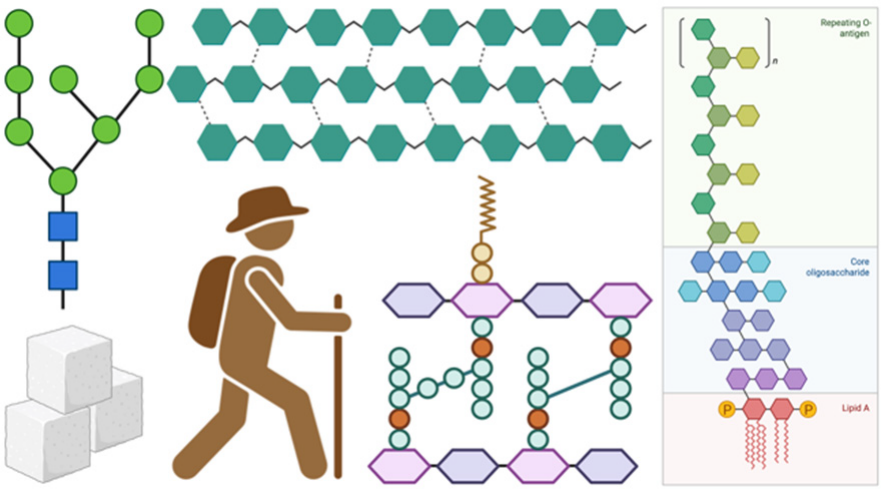

Oligosaccharides are ubiquitous in molecular biology and are used for functions ranging from governing protein folding to intercellular communication. Perhaps paradoxically, the exact role of the glycan in most of these settings is not well understood. One reason for this contradiction concerns the fact that carbohydrates often appear in heterogeneous form in nature. These mixtures complicate the isolation of pure material and characterization of structure–activity relationships. As a result, a major bottleneck in glycoscience research is the synthesis and modification of pure materials. While synthetic and chemoenzymatic methods have enabled access to homogeneous tool compounds, a central problem, particularly for newer synthetic chemists, is the matter of problem selection. This outlook aims to provide an entry level overview of fundamental principles in carbohydrate chemistry with an eye toward enabling solutions to frontier challenges.

## Abstract 2

A major bottleneck in glycoscience research is the synthesis and modification of pure materials. This outlook discusses fundamental carbohydrate synthesis from the purview of problems that are solved and issues that persist. Our goal is to inform new researchers to the field of key gaps in the field.

## Introduction

The programming and transmission of biological information is dependent on the construction of macromolecules that can encode the message. Carbohydrates, along with lipids, nucleic acids, and proteins are the four major classes of biomacromolecules found in eukaryotes and are responsible for information transfer.^1^ For example, DNA, which is a nucleic acid, stores the genetic information that cells need to synthesize proteins. A structurally related nucleic acid, RNA, exports the blueprint from the cell nucleus to the ribosome where protein synthesis occurs. Once produced, proteins catalyze most of the reactions in living cells. Each of these three biopolymers are produced in linear form under the guidance of a reliably templated biosynthesis. DNA and RNA are composed of four nucleotides, while mammalian proteins incorporate 20 proteinogenic amino acids. To contrast, carbohydrate biosynthesis is not templated, and molecules are composed of a variable number of monosaccharides which often only differ structurally by stereochemistry, ring size, and conformation.

**Figure 1:**
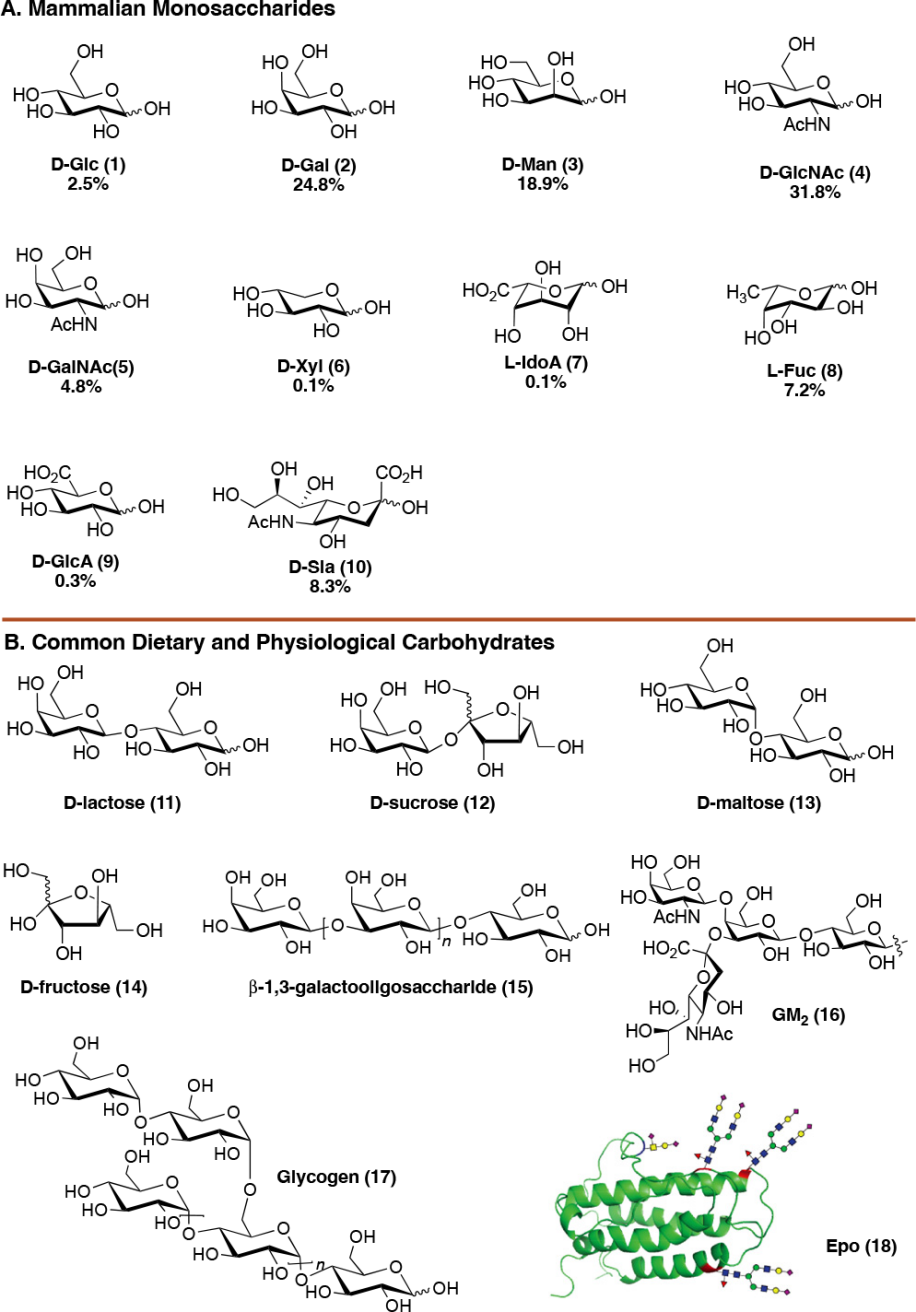
(A) Structures of the ten eukaryotic monosaccharides and their relative abundance. (B) Examples of common dietary and physiological carbohydrates and glycoproteins. Abbreviations: Glc, glucose; Gal, galactose; Man, mannose; GlcNAc, *N*-acetylglucosamine; GalNAc, *N*-acetylgalactosamine; Xyl, xylose; IdoA, iduronic acid; Fuc, fucose; GlcA, glucuronic acid; Sia, *N*-acetylneuraminic acid. The symbol nomenclature for glycans (SNFG) is a community-developed standard for simple representation of monosaccharides and glycans and was used to represent the glycans on EPO. GlcNAc (blue square), Man (green circle), Gal (yellow circle), Sia (purple diamond), Fuc (red triangle).

Most biomedical researchers are familiar with the 10 monosaccharides found in animals (**1**–**10**, Figure 1A). The relative abundance of mammalian monosaccharides is discussed in an important article on the “glyco-space” from the Seeberger lab.^2^ Outside of the field, however, people are most familiar with carbohydrates in the context of their diets and medications (**11**–**18**, Figure 1B). For example, carbohydrates are a primary source of nutrition as they are consumed in the form of fiber and starch. These complex carbohydrates are found in all grains and vegetables.^3^ Simple sugars are found mainly in fruits (glucose, sucrose, fructose) and milk (lactose). During digestion, the body metabolizes complex carbohydrates into simple sugars which enter the bloodstream and are transferred to individual cells to use for fuel in the form of glucose.^4^ Outside of their role in metabolism, carbohydrates rarely exist in nature as simple monosaccharides or disaccharides. Rather, they serve as building blocks for more complex macromolecules. For example, carbohydrates often exist as polymers where a series of monosaccharides are covalently linked through glycosidic bonds between the anomeric carbon of one sugar and an alcohol of another. The resulting molecules are categorized as either oligosaccharides (fewer than eight residues) or polysaccharides (greater than eight residues) based on the number of monosaccharides incorporated into their structure.^5,6^ An example of an oligosaccharide is a class of carbohydrates known as the galactooligosaccharides (GOS), **15**. These oligosaccharides are prebiotics composed of three to eight β-linked galactose residues.^7^ To contrast, an example of a polysaccharide is glycogen, **17**, which is the polymer found in the liver and muscles used to store excess glucose.^8^ Carbohydrates also exist in the form of glycoconjugates where they are attached to proteins or lipids.^9,10^ Well known examples are the gangliosides (such as GM_2_, **16**), which are glycosphingolipids highly abundant in the nervous system.^11^ Erythropoietin (EPO), **18**, is an example of a well-known glycoprotein which is secreted by the kidneys as a response to hypoxia.^12,13^ The glycans on EPO represent ca. 40% of the total mass of this protein, and it exists in nature as a mixture of several hundred glycoforms. Given its defined function, but imprecise structure, it is not surprising that EPO has been the topic of immense synthetic research.^14−20^

Given the different forms that carbohydrates exist as, carbohydrate complexity is expectedly intricate. First, carbohydrate structures can be linear or feature branching. Moreover, each new glycosidic bond creates a new stereocenter. Lastly, carbohydrate complexity is enhanced by the type of residue present, ring size (e.g., pyranose or furanose), and modifications such as sulfation, methylation, and phosphorylation. Given the volume of possible modifications, it is surprising that carbohydrate biosynthesis is not under genetic supervision. Rather, the biosynthesis of carbohydrates depends on a range of glycosyltransferases and glycosidases - enzymatic machinery responsible for forming and breaking glycosidic bonds.^21^ The action of these “self-governing” enzymes leads to the significant microheterogeneity and diverse structural motifs observed in naturally occurring oligo- and polysaccharides.

The increasing recognition of the roles of carbohydrates and their glycoconjugates in fundamental life processes has served to heighten the need for tractable quantities of material. As described previously, glycoconjugates are difficult to isolate in homogeneous form from living cells due to their microheterogeneity. Even when practical, the purification of carbohydrates from nature is typically tedious and low yielding. Thus, there remains a major opportunity for organic synthesis to provide key tools that will enable efforts across the glycosciences.

## Problem Selection

Ten years ago, the late Professor Gilbert Stork suggested to one of our authors that a common misconception among trainees in chemical synthesis is that all significant problems in the field have been solved. Contrarily, chemists are more likely to have been exhausted by the difficulty of the field’s central problems. This is why a diverse talent pipeline is critical: New practitioners in an established field can approach a problem without prejudice and will discover solutions to long-standing problems.^22−24^ With this concept in mind, our approach to this outlook is to describe several key roadblocks in carbohydrate synthesis in the context of community strengths and challenges. This outlook is organized as an introductory handbook for new practitioners as we discuss fundamental reactivity with an eye toward frontier areas where organic chemists can have an oversized impact.

### Manipulating Monosaccharide Building Blocks

Developing universal methods to synthesize monosaccharide building blocks for complex carbohydrate synthesis is a major challenge in the field. During a synthetic campaign, where monosaccharides are conjugated to generate larger structures, two key requirements must be addressed. First, alcohol and amine functional groups that are not modified in the final target must be temporarily protected. Second, the operator must decide at what point during the synthesis will modifications (deoxygenation, epimerization, etc.) and functional groups be installed. Ultimately, it is not until suitable building blocks are in hand, that a glycosylation reaction can occur.

Several important reviews have been curated to discuss the selective protection of carbohydrates.^25−27^ Historically, the basic premise is to leverage the inherent reactivity difference between alcohols on the sugar to achieve site-specific alkylation or acylation. Rather than recapitulate this coverage in full, our aim is to orient the reader to basic physical organic principles involving alcohol nucleophilicity as these principles are foundational to developing new strategies and tactics. Of note is that the coverage in this section is due to the great scholarship curated by, among others, Professor David Crich, Professor Todd Lowary, and the late Professor Bert Fraser-Reid.

#### Acetal and Ketal Formation

**Figure 2:**
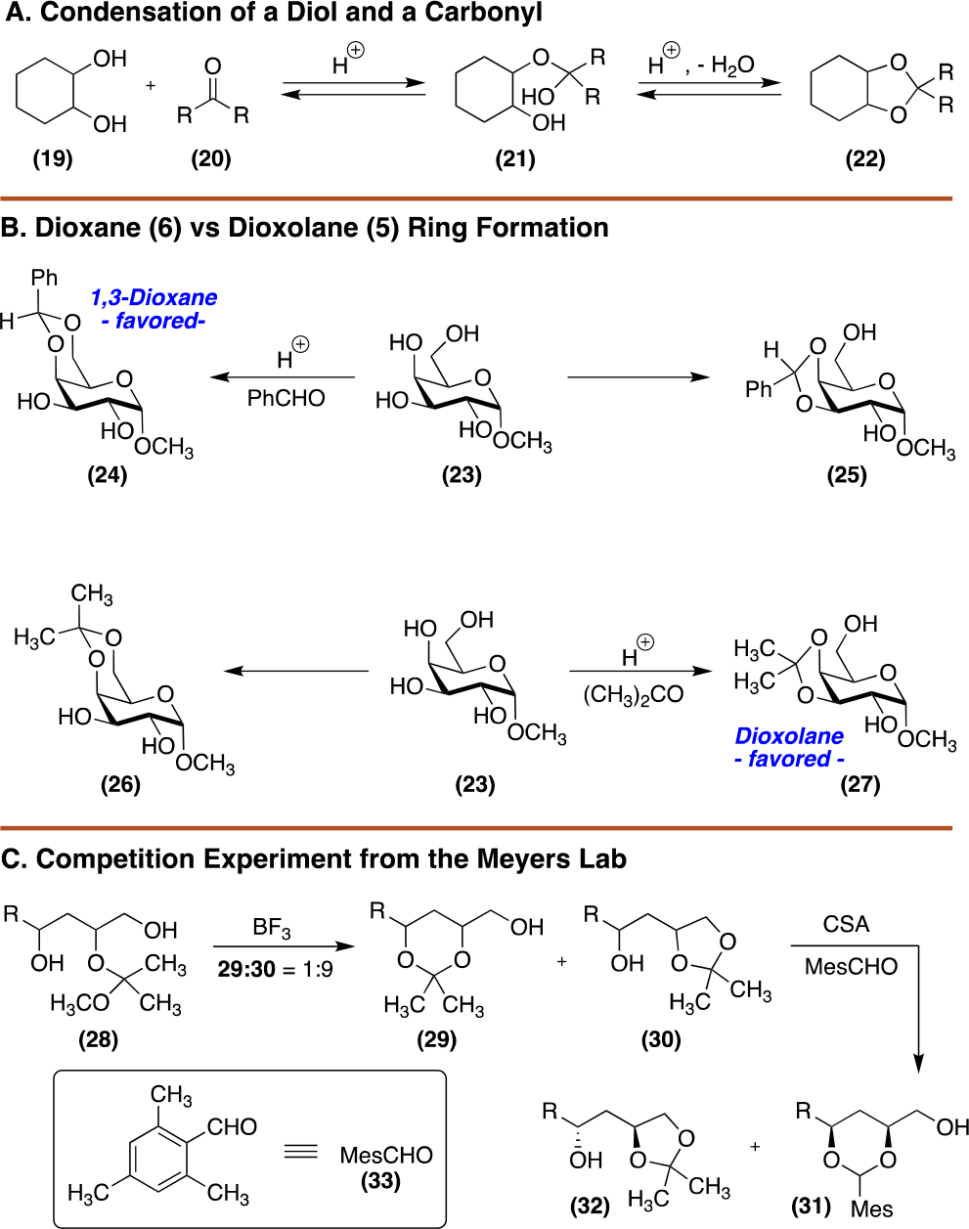
(A) Condensation of a diol and a carbonyl. (B) Aldehydes preferentially react with 1,3-diols to form dioxanes. Ketones preferentially react with 1,2-diols to form dioxolanes. (C) Competition experiment designed by the Meyers lab.

The condensation of a polyol with an aldehyde or ketone is one of the most well studied reactions in organic synthesis (Figure 2A).^28,29^ In the reaction, a Bronsted or Lewis acid catalyst promotes formation of an acetal or ketal, from the corresponding aldehyde or ketone. The product that forms depends on two factors: the stereochemistry of the alcohols involved in the forming cycle and the nature of the carbohydrate being condensed (furanose, pyranose, fully unprotected, etc.). While the product that is formed can vary widely, there are several general trends (Figure 2B). 1,3-Diols condense rapidly with aldehydes to form six-membered ring dioxanes (**23** to **24**), whereas 1,2-diols preferentially react with ketones to form five-membered ring dioxolanes (**23** to **27**).^30^ Mechanistically, the geminal substituents of a ketal cause severe 1,3-diaxial interactions in a six-membered ring, whereas these interactions are minimized in a five-membered ring. Additionally, 1,3-dioxane acetals can exist in a chair conformation with the acetal proton disposed in an axial position. While aldehydes condense fastest onto *cis*-1,3-diols, slower cyclizations are possible with *cis*-1,2-diols. In the context of monosaccharides, 1,3-dioxane formation occurs preferentially between O3 and O5 on furanosides and O4 and O6 on pyranosides. Each of these points were highlighted in the 1980s and 1990s by the Meyers lab in the context of their work on Streptogramin antibiotics (Figure 2C).^30^ Dioxane and dioxolane formation were evaluated in a competition experiment. When exposed to acidic conditions, an internal ketal **28** cyclizes such that a five-membered ring acetonide **30** is formed as the major product. Interestingly, if **30** is exposed to acid and aldehyde **33**, the *cis* 1,3-diol will be converted to the six-membered ring acetal **31**.

**Figure 3:**
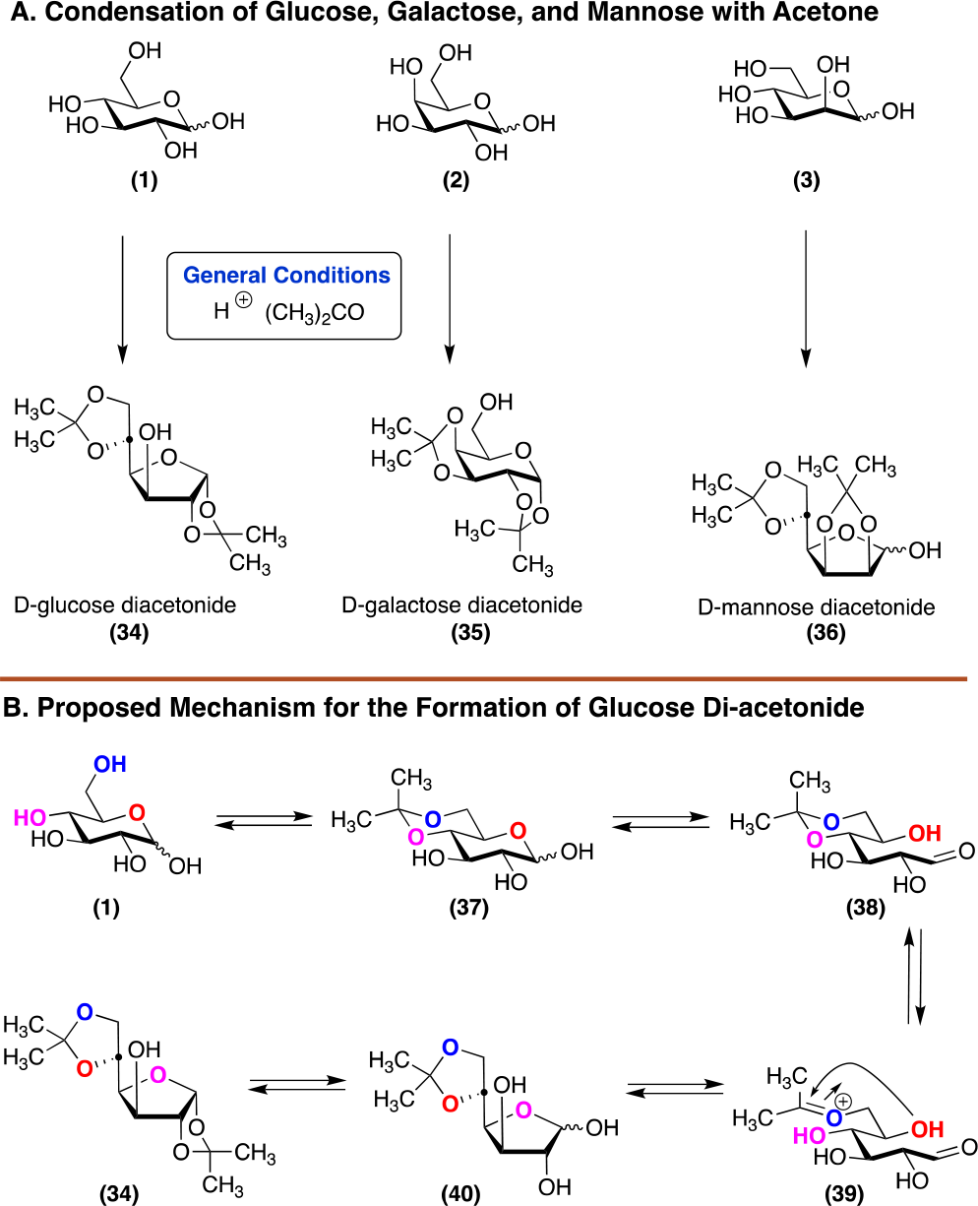
(A) Reaction of glucose, galactose, and mannose with acetone and acid. (B) While glucose exists primarily in its pyranose form, its condensation with acetone produces a furanose sugar. Oxygen atoms at C4 (purple), C5 (red), and C6 (blue) are highlighted to ease analysis of the abbreviated mechanism.

From a synthesis standpoint, the most useful acetal reactions involve isopropylidene (acetone) ketals and benzylidene (benzaldehyde) acetals. Figure 3 shows the thermodynamic isopropylidene products that are formed when acetone is condensed with glucose, mannose, and galactose. While simple on their surface, these reactions are mechanistically intriguing for several reasons. First, a monosaccharide will condense with as many equivalents of a carbonyl as possible which can lead to an unpredictable product distribution (Figure 3A). Second, isopropylidene formation appears to violate Baldwin’s rules as it must proceed via 5-*endo-trig* ring closure (see **39** to **40**). Third, thermodynamic products are favored, and the products produced are dictated by the relative stereochemistry of the alcohols present on the sugar. Though we are taught early on to visualize these monosaccharides in their pyranose forms, condensation with acetone will generate products with nonintuitive ring sizes (Figure 3B). For example, when glucose condenses with acetone, the kinetic acetonide is likely formed between O4 and O6. This molecule contains two *syn*-pentane interactions as one of the geminal methyl groups is disposed axially. At this stage, the substrate likely rearranges to its furanose form which features two five-membered ring acetonides. On a final note, forming acetals or ketals on glycosides proceeds with greater control of product distribution than reducing sugars since ring size is fixed.

#### Site-Specific Modifications

**Figure 4:**
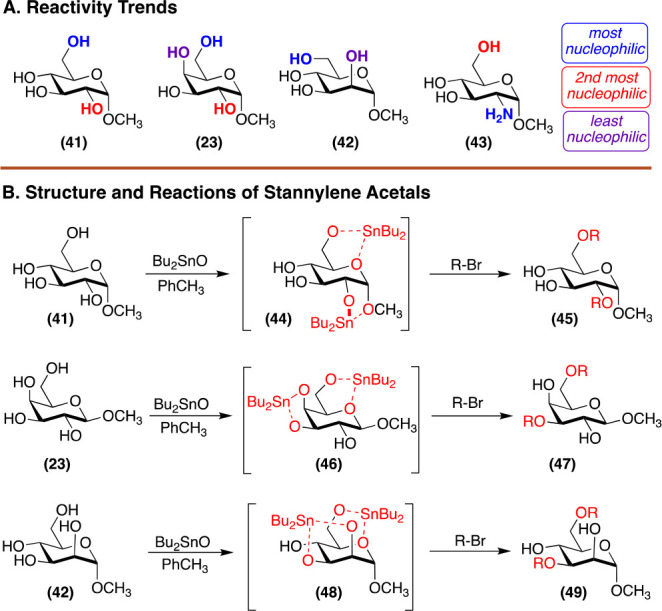
(A) General nucleophilicity trends. (B) Hypothetical structures and reactivity patterns of stannylene acetals.

Acetalization and ketalization are powerful tools as they can be used to protect multiple alcohols in a single reaction. Unfortunately, reactions of this type enhance concession steps.^31^ To avoid this scenario, site-specific alcohol modification has been an ongoing topic in carbohydrate synthesis (Figure 4). Since primary alcohols are less sterically hindered than secondary and tertiary alcohols, they can react selectively (Figure 4A). Steric hindrance can also be used to discriminate between two secondary alcohols. Generally, equatorial alcohols have greater nucleophilicity when they are adjacent to axial alcohols or ethers. Based on a steric argument, an equatorial alcohol adjacent to axial substituents would be more accessible than ones flanked by other equatorial substituents. Lastly, due to their enhanced nucleophilicity, amines can react preferentially in the presence of any alcohol regardless of its stereochemistry or configuration.

Based on the previous analysis, selective modification of C6 and equatorial alcohols in the presence of axial alcohols are straightforward. However, one will encounter issues when attempting to modify alcohols at C2, C3, or C4, particularly when each alcohol is oriented in an equatorial manner. To address such an issue, a popular method for regioselective reaction of 1,2 diols was developed by the Hanessian lab and is mediated by organostannane reagents such as dibutyltin oxide (Figure 4B).^32^ Exposure of glycosides to dibutyltin oxide generates an intermediate stannylene acetal under Dean–Stark conditions. These stannylene alkoxides are then treated with an electrophile to give the modified glycoside. Using this strategy, regioselective reaction with a wide range of electrophiles has been shown.^33−37^ Several trends can be predicted from this system. For *cis*-diols, the equatorial alcohol is functionalized.^38^ An alcohol next to a deoxygenated carbon has enhanced nucleophilicity.^39,40^ If the reacting diol is *trans* configured and has adjacent substituents, the alcohols are not easily distinguished.^41^ Contrastingly, if the *trans*-diols are adjacent to one axial and one equatorial substituent, selective protection of the equatorial alcohol next to the axial substituent will occur.^42^

**Figure 5:**
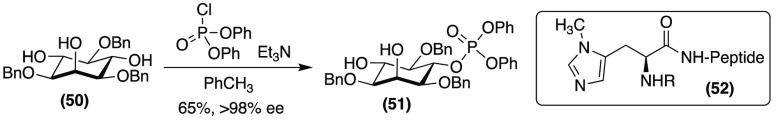
Asymmetric phosphorylation using a kinase mimic developed by Miller and colleagues.

While there are additional fundamental topics that warrant treatment, we close by discussing selective modification governed or influenced by biological systems. Not surprisingly, enzyme-catalyzed methods for selective modification of carbohydrates are well-described in the literature.^43^ We conclude, however, by discussing the elegant biomimetic logic developed by the Miller lab (Figure 5). An early development from the group was a “kinase-mimic” for asymmetric phosphorylation of *meso*-inositol derivative **50**.^44,45^ The catalyst, **52**, is a small peptide containing histidine residues which was found by screening an in-house library. **50** can undergo site-specific phosphorylation with excellent *ee* and good yield.

#### Functional Group Interconversion

**Figure 6:**
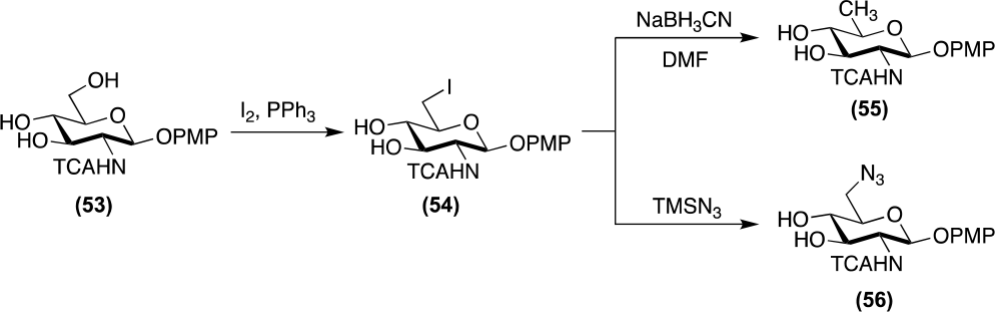
Example functional group interconversion via halide handles.

Conversion of an alcohol to its halide or pseudohalide is well studied.^46^ Like selective protecting group manipulation, regioselective iodination or bromination of the primary alcohol at C6 is facile and can be achieved on minimally protected glycosides. Polyhalogenation is also possible under specific conditions.^47^ Strategically, glycosyl halides are used for two purposes (Figure 6). The first is nucleophilic substitution chemistry where, for example, glycosyl azides can be prepared by reaction of the halide with NaN_3_ or TMSN_3_.^48^ The azide can be used as a masked amine or further leveraged in click chemistry.^49^ The second tactic is as a selective handle for reductive dehalogenation to generate deoxy sugars or epimerization of secondary alcohols.^50,51^

#### Challenge: Synthesis of Differentially Protected Building Blocks

Although masterful applications of physical organic chemistry, any series of protecting group manipulation will hamper material throughput. This is a central reason why many carbohydrate syntheses are long. A major advance is more efficient chemical methods to access a core set of differentially protected carbohydrate building blocks. *Building block synthesis, rather than chemical glycosylation, is the major bottleneck in the chemical synthesis of oligosaccharides.* While some building blocks are available for mammalian sugars, nonmammalian monosaccharides are not commercially available. Currently, their preparation can be completed using one of three approaches. The first is the modification of readily available monosaccharides.^52^ This can be advantageous as the starting material is chiral. Unfortunately, syntheses require extensive protecting group manipulation. The second approach is *de novo* synthesis from noncarbohydrate starting materials in the chiral pool, an approach that shortens routes.^53−56^ Moreover, this strategy is the best method to prepare extensively deoxygenated or functionalized monosaccharides. Lastly, advances in asymmetric catalysis have enabled several elegant syntheses of carbohydrate building blocks from nonchiral starting materials.^57^

**Figure 7:**
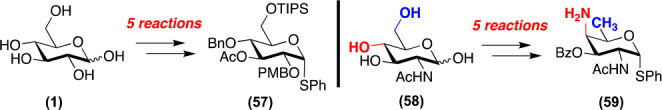
Wish list for building blocks and direct point mutations.

In addition to new methods to rapidly access building blocks, it would be transformational if the field could access all mammalian monosaccharides as building blocks of type **57** (Figure 7). Ideally, these building blocks would be prepared in a step count that equals the number of modifications. For example, is it possible to convert glucose **1** to **57** such that a latent leaving group is placed at C1, alongside four orthogonal protecting groups at C2, C3, C4, and C6 using just five reactions? This type of innovation for all 10 mammalian monosaccharides would rapidly enable the synthesis of oligosaccharides.

#### Challenge: Direct Functional Group Interconversion

**Figure 8:**
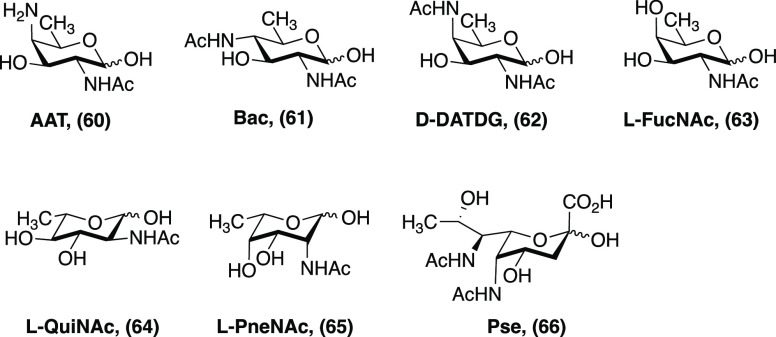
Examples of bacterial monosaccharides. Abbreviations: AAT, 2-acetamido-4-amino-2,4,6-trideoxy-d-galactose; Bac, bacillos-amine, DATD, 2,4-diacetamido-2,4,6-trideoxy galactose; l-FucNAc, *N*-acetylfucosamine; l-QuiNAc; *N*-acetyl-l-quinovosamine; l-PneNAc, *N*-acetyl l-pneumosamine; Pse, pseudaminic acid.

The challenges, however, do not stop at the mammalian glycome. While eukaryotes produce ten sugars, bacteria produce hundreds more, most of which are significantly more functionalized (Figure 8). Access to these molecules will depend on innovations to *de novo* synthesis and feedstock carbohydrate modification.^58,59^ Previously, our group and others have focused on methods to synthesize 2-acetamido-4-amino-2,4,6-trideoxy-d-galactose, colloquially known as AAT, **60**. Each synthesis is arduous and generally ranges from 10 to 15 steps.^50,60−62^*If the technology were available to deoxygenate carbon atoms site specifically and convert alcohols directly to amines (with either retention or inversion of stereochemistry), synthesis of bacterial monosaccharides would be significantly enhanced.*^63^

#### Challenge: Glycosylation

**Figure 9:**
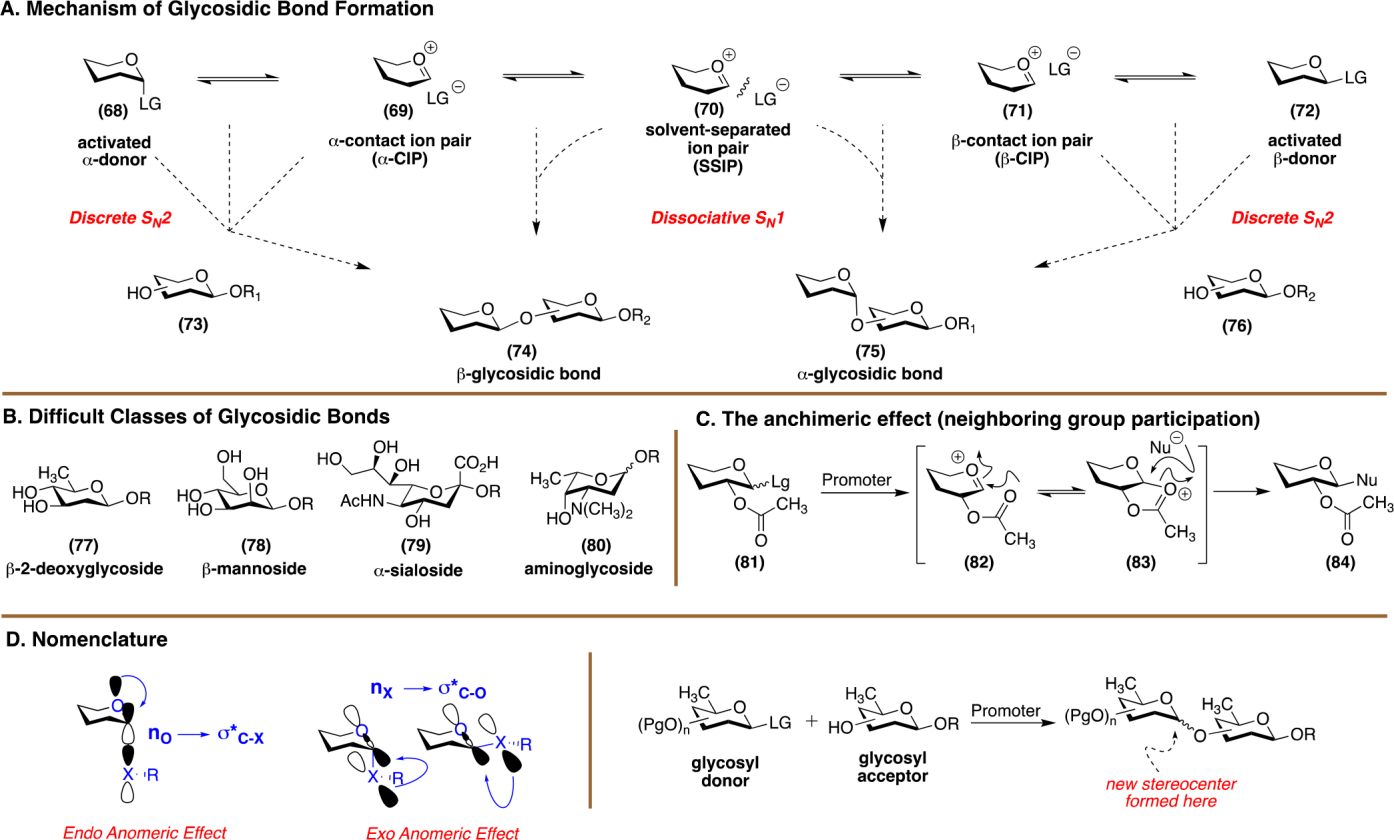
(A) An abbreviated mechanism of chemical glycosidic bond formation. (B) Difficult classes of glycosidic bonds. (C) A visual representation of neighboring group participation. (D) Useful glycosylation nomenclature.

Glycosidic bond formation involves transformation of a sugar into a fully protected glycosyl donor with a latent leaving group at its anomeric center (Figure 9A). Once activated, the donor reacts with a suitably protected acceptor. In the reaction, the donor functions as the electrophile while the acceptor functions as the nucleophile. The latent leaving group and the protecting group patterns on the substrate are the most important parameters that influence the yield and diastereoselectivity of a glycosylation reaction. Other conditions, such as concentration, temperature, and order of reagent addition also influence diastereoselectivity. Mechanistically, glycosylation is a nucleophilic substitution reaction, since the reaction is centered on displacement of a leaving group. As extensively described by Crich, Lemieux, and many others, two pathways are at play: a unimolecular, dissociative S_N_1 process involving a discrete oxonium ion and a bimolecular S_N_2 pathway advancing through an associative transition state. These pathways span a range of ion-pair mechanisms, i.e., covalently bound donors in equilibrium with their contact ion pair and solvent-separated ion pair.

In 2011, Professor Crich authored an important article where he described how a combination of methodology development with an in depth understanding of physical organic chemistry could rapidly advance carbohydrate science.^64^ He noted that, of the different classes of glycosidic bonds, several are difficult to form with absolute stereocontrol (Figure 9B). For example, α-glucosides, α-sialosides, β-mannopyranosides, and all deoxy sugars, particularly 2-deoxy-α-glycosides, are difficult bonds to install. α-Glucosides are challenging since they cannot be readily prepared by leveraging anchimeric assistance from C2 (Figure 9C). The remaining are equatorial glycosides which are thermodynamically unfavorable due to the lack of negative hyperconjugation (Figure 9D). Two of the three bonds are deoxy sugars, which is accompanied by enhanced activation toward oxocarbenium ion formation. An equally formidable, yet less appreciated, challenge is the stereoselective construction of a glycoside where the donor or acceptor incorporates a basic amine that can engage the promoter; consider this as promoter poisoning. Thus, traditional amine-containing glycosyl donors and acceptors must either feature a masked amine with specific electronic and steric properties or proceed via a noncationic mechanistic pathway.^65^*While conceptually difficult, a general set of reaction conditions that can enable the formation of any glycosidic bond with complete diastereocontrol would transform carbohydrate synthesis.* A last important point of discussion is glycosylation of unprotected sugars. Selective glycosylation of unprotected sugars is a particularly significant goal, requiring not only control over diastereoselection but also differentiation between similarly reactive alcohols. Going forward, we believe the important innovations in this area will come from synthetic biologists and organic chemists with knowledge of chemoenzymatic approaches.^66^

#### Challenge: Automated Carbohydrate Synthesis

The advances described in the sections above have contributed significantly to programmable methods for oligosaccharide synthesis analogous to those used in peptide synthesis.^67−75^ To preface, the synthesis of linear peptides is largely automated, though with size limitations. Technologies such as native chemical ligation, however, assist in mitigating this issue.^76−80^ Progress in the synthesis of peptides is the result of a remarkable understanding of the physiochemical properties governing their assembly. To a similar end, characterizing the parameters to achieve automated chemical assembly of oligosaccharides is of ongoing interest.^67,81,82^ In 2011, the Wong lab published an outstanding review describing the development of automated oligosaccharide synthesis.^83^ Interestingly, most approaches, particularly one-pot approaches,^84^ leverage orthogonally protected thioglycoside donors of different reactivity. Thioglycosides are generally bench stable and can be activated under a wide range of conditions.^85−90^ Much like peptide synthesis, the potential of automated synthesis appears to be limitless. For example, in 2013 the Seeberger group demonstrated the synthesis of a 30-mer mannoside.^91^ Less than a decade later, in 2020, the group demonstrated the synthesis of a 151-mer polysaccharide.^92^ While additional glycans of considerable length have been generated, we note that the Ye lab synthesized a polysaccharide incorporating 1080 residues using automated technology.^93^ It is important to note that while the community is pushing the limitations of automated synthesis, most targets contain only alcohol functionality and thus preclude a number of bacterial targets. Development of the necessary building blocks and universal conditions for automated synthesis of targets such as capsular polysaccharides would greatly enable the study of their biochemical properties.

#### Challenge: Carbohydrate conjugation to proteins

Glycosylation patterns of proteins and lipids serve as recognition elements for various cellular processes and interactions. For example, lipopolysaccharides are an important component of cell membranes of gram-negative bacteria and elicit an innate immune response in vertebrates. Similarly, host cells present glycolipids associated with self as part of immune system regulation.^1^ Fully mature proteins exist with several glycosylation patterns giving rise to different glycoforms. *However, a nuanced understanding of the effects of different glycosylation patterns is a fundamental challenge yet to be met.* Isolation of single glycoforms from natural sources has proved poor yielding and impractical using current methods. Thus, accessing glycoforms by synthetic or semisynthetic means is an attractive alternative. Indeed, synthesis can be used to generate glycoproteins with unnatural patterns and is a promising strategy to manipulate their physicochemical properties and bioavailability.^94^ This section will focus on current and future directions for the synthesis of glycoproteins.

Challenges encountered in the synthesis of protein glycoconjugates are dependent on the size of the target. Natural glycoproteins can be broadly classified as N-linked or O-linked. N-Linked glycoproteins feature conjugation of the sugar to the amide side chain of asparagine (Asn) residues. O-linked glycoproteins incorporate glycans on the alcohol side chains of serine (Ser) or threonine (Thr) residues. Fmoc solid-phase peptide synthesis (Fmoc-SPPS) can be automated and, importantly, the deprotection/cleavage conditions are compatible with O- and N-glycosylated peptides, though not without caveats.^59^

Chemical glycosylation of mature peptide chains is often low yielding due to the low reactivity of the alcohol side chains of Thr and Ser residues. For this reason, the more common strategy is the incorporation of glycosylated amino acids during SPPS. If low molecular weight glycopeptides (generally <50 amino acids) are the target, then much of the challenge is in synthesizing glycosylated amino acid building blocks. For glycoproteins (>50 amino acids), coupling of smaller peptide fragments presents an additional challenge.

Native chemical ligation (NCL) of glycosylated peptides is the current method of choice for synthesis of glycoproteins. Initially reported by Kent in 1994,^79^ the scope of NCL has since been enhanced by several enabling methodologies such as ligation at noncysteine residues, ligation with selenium containing amino acids, and metal-free chalcogen removal. These methods have enabled synthetic access to homogeneous glycoproteins.^95−97^ These advances in NCL are discussed in recent reviews.^98,99^ In addition, the Muir lab has published work on split inteins as a method for expressed peptide ligation.^100,101^ Using flow chemistry, exceptional work in the Pentelute lab demonstrates automated synthesis of peptides up to 164 amino acids in length.^102^ Looking to the future, application of these advances to the synthesis of glycoproteins would be highly enabling.

**Figure 10:**
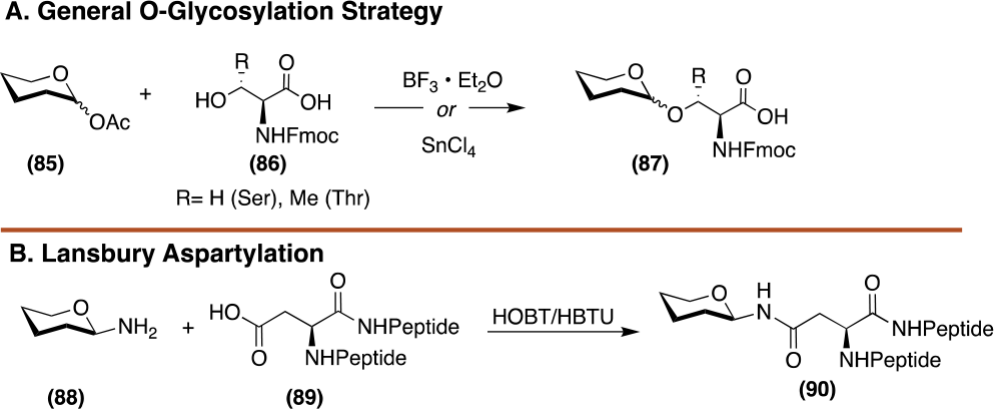
General strategies for O*-* and N-glycosylation.

As described above, O-glycosylation of Ser or Thr residues and N-glycosylation of Asn residues are naturally occurring patterns. These amino acids are commercially available as glycosylated building blocks in protected forms. The synthesis of so-called O-linked amino acid “cassettes” has been demonstrated using glycosyl halides or acetates as donors under Lewis acid conditions (Figure 10 A).^103−105^ Asn-glycosylation is accomplished via Lansbury aspartylation where a glycosyl amine is coupled to aspartic acid using carbodiimide activation (Figure 10 B).^106,107^ Unfortunately, these building blocks are expensive and often cost ca. $500 per gram. *This financial barrier can be alleviated with methodology for facile access to these glycosyl amino acids and prove vital to the iterative approach to glycopeptide synthesis.*

We would be remiss not to mention how this method would also affect oligosaccharide incorporation into glycoproteins. There are two common ways of incorporating an oligosaccharide of interest into a glycoprotein: (1) Glycosylation of a constituent polypeptide before NCL and (2) enzymatic elaboration of a sugar handle to the desired oligosaccharide after NCL.^103^ The second approach is more synthetically attractive due to late-stage incorporation of acid sensitive glycans. Overall, the total synthesis of glycoproteins is a formidable challenge in organic synthesis. New advances to carbohydrate-amino acid conjugation methodologies will continue to enable the generation of glycosylated proteins with predesigned modifications.

#### Challenge: Vaccination Using Carbohydrate Antigens

Glycans are directly involved in normal physiology and in the etiology of every major disease afflicting humankind. Accordingly, characterizing the cell surface glycome creates an expanding frontier for improving human health and wellness. In this section, we describe the roles glycans play in fundamental biological processes, such as inflammation and immune system activation. We then discuss frontier problems concerning vaccine development in the areas of infectious diseases and cancer. We close by discussing the role of glycans in the development of new agents of therapeutic value.

##### Infectious Diseases

Trillions of microbial cells exist within and on the human body (at a ratio close to 1:1).^108^ While most members of the flora are harmless, infectious diseases caused by bacteria, viruses, and parasites are a health problem encountered by all global communities. Breakthroughs used to mitigate and control infection have occurred through two processes. The first is the development and application of antimicrobial agents, antibiotic, antiviral, and antiparasitic compounds, which actively inhibit disease pathogenesis. In addition to small molecule therapeutics, vaccines were developed to protect against serious infectious diseases. For example, while one of our authors dealt with chickenpox as a child in 1991, all remaining authors were born after 1995 when the chicken pox vaccine was developed and are part of the 90+ million cases that have been prevented. Vaccination is the most cost-effective method to prevent major illness due to infectious diseases. Indeed, the development of effective vaccines against diseases caused by viruses, parasites, and bacteria is arguably more urgent now than at any time since the introduction of the polio and smallpox vaccines.

So called “whole cell” vaccines were the first vaccines developed by humankind, consisting of attenuated or dead microorganisms.^109^ While whole cell vaccines offer long-lasting immunity against infectious diseases, two issues complicate their use. First, production is challenging particularly at large scale. Second, weakened pathogens can cause disease in immunocompromised people.^110^ As an alternative, subunit vaccines were introduced. Rather than using the entire pathogen, subunit vaccines include only antigenic components from the cell which are most capable of stimulating the immune system. Traditionally, the favored component of subunit vaccines has been proteins due to their ability to induce a robust immune response.^111^ Protein antigens, however, are not without challenges as they can fail to illicit an immune response. For example, there are a wide range of cell surface proteins all of which are not sufficiently immunogenic. Carbohydrate-based antigens have long been viewed as an alternative to protein antigens.^112−120^ For example, successful glycoconjugate vaccines made from isolated capsular polysaccharides have been developed.^121^ As these materials are typically poorly immunogenic, subunit vaccines are usually formulated with adjuvants.^122−126^

Presently, carbohydrate-based vaccines are used in the clinic to protect patients from several viruses (*Haemophilus influenza* type and *Streptococcus pneumoniae*). While these vaccines are a success, developing new carbohydrate-based vaccines continues to be challenging. Key gaps that can be solved by new practitioners are two-fold. *First is enabling access to defined synthetic oligosaccharide antigens. The second advance is deciphering how to correctly present vaccine candidates to the immune system to elicit a robust response*. Though an intimidating area, new practitioners to the field can turn to the Seeberger lab for an example of the endless possibilities available in the area. Indeed, the lab has developed several synthetic carbohydrate vaccine constructs that are either fully synthetic or produced through semisynthesis. These conjugates were active against a wide range of pathogens, including *Acinetobacter baumannii*,^127,128^*Clostridium difficile*,^129^*Klebsiella pneumoniae*,^130^ and *Streptococcus pneumoniae*.^131−135^

##### Cancer

Among strategies to eradicate cancerous cells, mobilizing the immune system is most appealing. Malignantly transformed cells express irregular levels and types of carbohydrates, a feature which differentiates tumor cells from healthy cells. A long-standing question in the community is whether it is possible to design vaccine constructs integrating these tumor-associated carbohydrate antigens (TACAs). If so, proper presentation of the vaccine to the immune system would induce a selective immune response, leading to a purging of tumor cells that overexpress the epitope. Unfortunately, no fully carbohydrate-based anticancer vaccine has progressed out of clinical trials, and this represents a major gap in the field.

The Danishefsky laboratory was an early pioneer in this area and explored the development of several vaccine formulations. For example, early work from the group focused on monovalent vaccines which were designed to immunize against a single antigen. These constructs included a single carbohydrate antigen attached to a carrier protein. One of the earliest monovalent approaches reported by the lab was the construction of Lewis (b and y) and H-type (I and II) blood group determinants which was inspired by Bernstein and Hall. The synthesis of this construct was enabled by the glycal assembly process - what was then a key development in carbohydrate synthesis methodology.^136^ However, one of the disadvantages of this approach is that it does not account for the actual degree of diversity of the carbohydrate epitopes that are expressed on a transformed cell surface. This shortcoming led to the development of a polyvalent approach which aimed to immunize against two or more antigens and thus had the possibility of targeting more than one cancer type. While the vaccine candidates could be produced, the approach had significant limitations, including a need to increase the levels of the carrier proteins, a validation tactic for the individual monomeric component of the polyvalent conjugate, and last, its low yields for conjugation to the carrier protein.

These two failed approaches birthed the unimolecular multivalent approach. This approach allowed for several carbohydrate antigens to be on a single polypeptide backbone. The first-generation approach was a unimolecular pentavalent construct containing five different prostate and breast cancer associated carbohydrate antigens (Globo-H, Le^y^, STn, TF and Tn) which were attached to a single peptide backbone. The entire glycopeptide was conjugated to a keyhole limpet hemocyanin (KLH). In mouse immunization studies, these KLH conjugates were observed to elicit a robust immune response.^137−139^

Another major contributor to cancer vaccine candidate development has been the Boons lab. The team synthesized anticancer vaccines that were composed of (1) tumor-associated Tn antigen; (2) a peptide, T epitope YAF, which succeeds in overcoming the T cells independent properties of the carbohydrate antigen, and (3) a lipopeptide, Pam_3_Cys, an immunoadjuvant, that has been shown to upregulate the production of cytokines and chemokines. This design triggers the production of antigen-presenting cells, leading to T-cell development and activation.^140^ The lab designed a robust three component vaccine composed of a TLR2 (Toll-like receptor) agonist, a peptide T-helper epitope, and a tumor associated glycopeptide which elicits a high IgG antibody response. They found that the immunoadjuvant TLR2 agonist, Pam_3_CysSK_4_, when attached to the B and Th epitopes allows for cytokines to be produced where the vaccine interacts with the immune cells. Therefore, leading to a high local concentration of cytokines.^141^

Though many other groups have contributed to this area,^142−151^ we close with a discussion on efforts from the Kunz lab which have primarily focused on the construction of anticancer vaccines with the incorporation of tumor-associated mucin (MUC1). One of the early strategies that was presented by the lab is the combination of the tumor associated sialyl Tn MUC1 glycopeptide antigen with a T-cell epitope of tetanus toxin using a flexible spacer.^152,153^ While much success has been achieved, characterizing the best methods to present carbohydrates to the human immune system such that a potent response is elicited remains a major challenge.

Taken together, developing vaccines against TACAs is an ongoing challenge. However, analysis of the combined efforts of several programs shows that the use of appropriate adjuvants, immunogenic carrier molecules, and the use of multivalency (multiple TACAs) may eventually lead to a functioning vaccine candidate.

## Conclusion

As Professor Nicola Pohl stated in a recent editorial: “Chemistry is now often the bottleneck to the development of a sophisticated understanding and use of this class of biomolecules as was true for nucleic acids and proteins before the invention of tools and techniques such as the polymerase chain reaction and automated solid-phase synthesis”.^154^ Our hope for this outlook is several-fold and really speaks to Professor Pohl’s thoughts. First, we hope that this introductory guide showcases the intriguing physical organic principles central to the development of new reactions in carbohydrate science. Second, there are many unmet needs at the frontier of carbohydrate science (analytical, chemical, and biological) that await new talents and new perspectives. Lastly, carbohydrates are central to several fields in the biological sciences. The frontier of discovery awaits those who can bring novel tools to address problems in areas ranging from infectious diseases and cancer to the microbiome and materials science.
